# Conditional expression of Spry1 in neural crest causes craniofacial and cardiac defects

**DOI:** 10.1186/1471-213X-10-48

**Published:** 2010-05-11

**Authors:** Xuehui Yang, Sean Kilgallen, Viktoria Andreeva, Douglas B Spicer, Ilka Pinz, Robert Friesel

**Affiliations:** 1 Center for Molecular Medicine, Maine Medical Center Research Institute, Scarborough, ME 04074, USA; 2 Department of Dental Research, Tufts University School of Dental Medicine, Boston, MA 02111, USA

## Abstract

**Background:**

Growth factors and their receptors are mediators of organogenesis and must be tightly regulated in a temporal and spatial manner for proper tissue morphogenesis. Intracellular regulators of growth factor signaling pathways provide an additional level of control. Members of the Sprouty family negatively regulate receptor tyrosine kinase pathways in several developmental contexts. To gain insight into the role of Spry1 in neural crest development, we analyzed the developmental effects of conditional expression of Spry1 in neural crest-derived tissues.

**Results:**

Here we report that conditional expression of Spry1 in neural crest cells causes defects in craniofacial and cardiac development in mice. *Spry1;Wnt1-Cre *embryos die perinatally and exhibit facial clefting, cleft palate, cardiac and cranial nerve defects. These defects appear to be the result of decreased proliferation and increased apoptosis of neural crest and neural crest-derived cell populations. In addition, the domains of expression of several key transcription factors important to normal craniofacial and cardiac development including *AP2*, *Msx2*, *Dlx5*, and *Dlx6 *were reduced in *Spry1;Wnt1-Cre *transgenic embryos.

**Conclusion:**

Collectively, these data suggest that Spry1 is an important regulator of craniofacial and cardiac morphogenesis and perturbations in Spry1 levels may contribute to congenital disorders involving tissues of neural crest origin.

## Background

Neural crest cells (NCC) are pleuripotent cells that migrate out of the dorsal neural tube during early vertebrate embryogenesis to populate many anatomical structures along the dorsoventral axis [1,2]. Cranial NCC migrate ventrolaterally from the forebrain and hindbrain region to populate craniofacial structures and branchial arches. The proliferation of cranial NCC results in a demarcation of each branchial arch. Once migration is complete, cranial NCC contribute to the maxilla, mandible, cranial ganglia, and other mesenchymally derived structures of the head and neck. Cardiac NCC emanating from rhombomeres 6-8 populate branchial arches 3, 4, and 6. Some cardiac NCC contributes to the development of the branchial arch arteries, cardiac outflow tract, and the spiral septum between the ascending aorta and the main pulmonary artery. Other cardiac NCC contribute to the formation of the outflow tract cushions/endocardial cushions and subsequently the semilunar valves and interventricular septum. Perturbations in normal neural crest development cause several congenital craniofacial and cardiac defects.

Cell-cell and tissue interactions are required for proper patterning of neural crest-derived structures. Several growth factors are important to NCC formation, migration, and differentiation, including members of the FGF family and their receptors [1,2]. The identification of mutations in fibroblast growth receptors (FGFRs) that cause several craniosynostosis syndromes indicates a role for FGF signaling in the skeletogenic differentiation of NCC [3,4]. Furthermore, NCC proliferate, migrate, and differentiate into cartilage and bone in vitro in response to FGF2 [5,6]. In addition, tissue-specific deletion of FGF8 demonstrated a requirement for FGF8 in NCC cell survival and patterning of the first branchial arch [7]. A hypomorphic allele of *Fgfr1 *has been used to demonstrate that FGFR1 is required for NCC migration into the second branchial arch [8]. Mice carrying this allele showed severe abnormalities of the craniofacial bones and cartilage. These and other studies show that FGF signaling is important to craniofacial development and that gene dosage in components of the FGF pathway is important to normal craniofacial development.

Sprouty (Spry) was originally identified in *Drosophila *as a negative regulator of FGF signaling in tracheal development [9]. Subsequently, Sprouty was demonstrated to inhibit EGF signaling in *Drosophila *eye development [10,11]. In vertebrates, there are four Sprouty proteins that either inhibit or potentiate receptor tyrosine kinase (RTK) signaling in a context specific manner [12,13]. For example, Spry2 can potentiate EGFR signaling by binding to c-Cbl and sequestering it away from the EGFR, thus preventing EGFR down regulation and degradation, consequently leading to sustained EGFR activation, and enhanced ERK signaling. Conversely, Spry2 inhibits ERK activation mediated by FGFR signaling. Thus, Spry proteins exhibit differential effects depending upon the cellular context.

During vertebrate development, Spry proteins exhibit overlapping patterns of expression, particularly in craniofacial structures and limb buds [14]. Gene targeting studies have revealed both distinct and redundant functions for Spry proteins during development. Targeted deletion of *Spry2 *results in defects of inner ear and in tooth development [15,16]. Deletion of *Spry1 *results in defects in kidney development where supernumerary branching of the ureteric buds occurs resulting in multiple ureters [17]. *Spry4 *null mice show defects in development of the mandible, polydactyly, and small size [18]. Mice that are null for both *Spry2 *and *Spry4 *alleles exhibit very severe craniofacial defects and dwarfism [18]. In addition, mice homozygous for a 1 MB deletion of chromosome 14, a region that encompasses the *Spry2 *gene, exhibited cleft palate and cleft lip of variable penetrance [19]. Interestingly, a mouse carrying a Spry2-BAC transgene rescued the cleft palate defect. However, the Spry2-BAC transgenic line expressed Spry2 at reduced levels suggesting that palate development is *Spry2 *dosage sensitive [19].

Due to the complex nature of Spry function and the possible redundancies during development, we developed a conditional *Spry1 *transgenic mouse. To investigate the role of Spry1 in regulating NCC during development, we induced tissue-specific expression of Spry1 using *Cre/loxP *recombination in the neural crest lineage by using *Wnt1-Cre *transgenic mice [20]. Our study shows that Spry1 expression in Wnt1-expressing neural crest cells in vivo results in facial clefting, cleft plate, failure of formation of the nasal and frontal bones as well as cardiovascular defects including ventricular septal defects, and outflow tract defects. Mutant embryos also exhibited hypoplastic thyroid, thymus, and cranial ganglia. Spry1 expression in NCC cells resulted in decreased proliferation and increased apoptosis. We conclude that Spry1 is a regulator of NCC cell proliferation and survival and that this occurs in both NCC cells and NCC-derived mesodermal cells that result in craniofacial and cardiac structures.

## Results

### Spry1 is expressed in migrating and post-migratory neural crest cells

**Figure 1:**
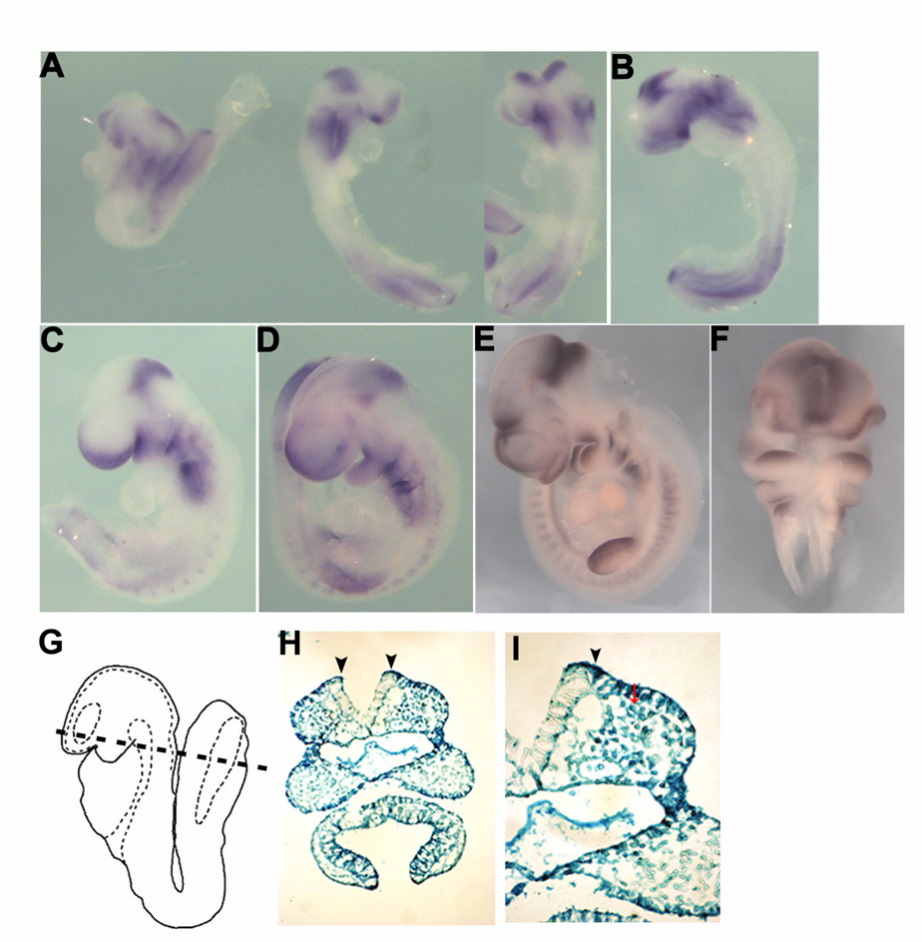
**The whole mount expression pattern of Spry1 in developing mouse embryos**. (A) E8.0, (B) E8.5, (C) E9.0, (D) E9.5 and (E, F) E10. Spry1 is expressed in the primitive streak, brachial arches, midbrain-hindbrain boundary, lateral mesoderm and tail bud. E8.5 *Spry1+/-LacZ *embryos were stained with β-gal and sectioned through the plane indicated (G). β-gal staining is evident in the presumptive neural crest (H, arrowheads indicate the neural folds) and higher magnification (I).

We examined the expression Spry1 in mouse embryos from E8.0 to E10.0 using whole-mount in situ hybridization. In situ hybridization analysis at E8.0 revealed that Spry1 is highly expressed in the cranial neural folds and presomitic mesoderm (Figure 1A) and continues to be expressed in regions populated by cells of neural crest origin including the branchial arches 1, 2, and 3, the frontonasal process, the midbrain hindbrain boundary, as well as limb buds and presomitic mesoderm at E9.0 (Figure 1C). This pattern persists until about E10.0 (Figure 1E,F). These expression data are consistent with a previous report [14]. To gain additional insight into the pattern of embryonic Spry1 expression, we performed β-gal staining on *Spry1*^*lacZ*/+ ^embryos at E8.5. A coronal section through the rostral region indicates β-gal staining in the presumptive neural crest (Figure 1H, I black arrowhead) as well as the underlying mesoderm (Figure 1I, red arrow).

### Conditional expression of Spry1 in neural crest cells

In situ hybridization and β-gal staining patterns of *Spry1 *suggests that it plays a role in the development neural crest derived sutures. To investigate this further we used transgenic mice with a floxed *mSpry1 *transgene, which efficiently undergoes Cre-mediated recombination as we have previously demonstrated [21]. To enable tissue specific expression in neural crest, we crossed *CAGGFP-Spry1 *transgenic females with male *Wnt1-Cre *transgenic mice. Bitransgenic mouse embryos were designated *Spry1;Wnt1-Cre *and were confirmed by genotyping for GFP and Cre by PCR [21]. Littermates carrying the *Spry1 *transgene, but lacking the Wnt1-Cre transgene served as controls in these studies. All *Spry1;Wnt1-Cre *mutant embryos died at birth and exhibited severe craniofacial defects (data not shown). Prenatal lethality was not observed. Attempts to show transgenic protein expression by immunohistochemistry proved difficult due to issues of sensitivity and background of the anti-myc antibody used to detect the epitope tagged transgenic protein. However, we have previously demonstrated Spry1 transgenic protein expression using the same transgenic mice when crossed with other transgenic Cre driver strains [21]. qPCR analysis revealed expression levels of the transgene to be 2-8 fold above endogenous expression levels (Yang, data not shown).

### *Spry1;Wnt1-Cre *embryos exhibit craniofacial defects

**Figure 2:**
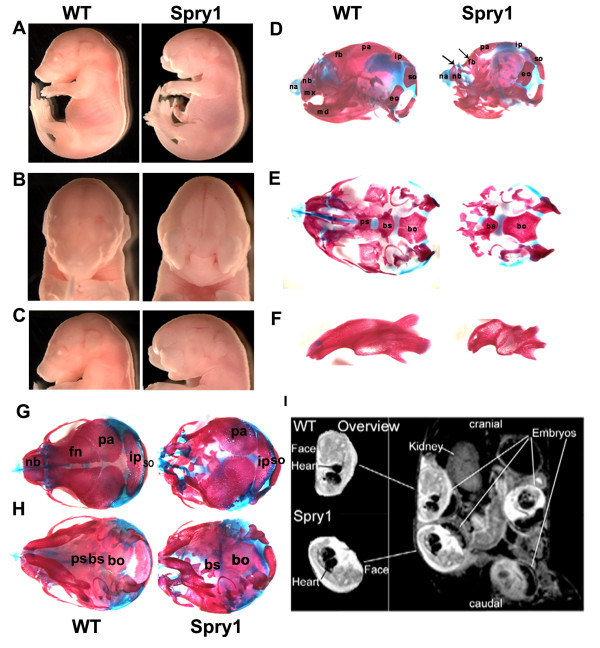
***Spry1;Wnt1-Cre *embryos exhibit craniofacial defects**. (A-C) E16.5 embryo imagines, (A) Lateral view of whole embryos (from original 1×), (B) frontal and (C) lateral views of head (from original 2.5 ×) shows facial clefting in *Spry1;Wnt1-Cre *(Spry1) embryos. (D-H) Skeletal preparations of E16.5 embryos stained by alcian blue and alizarin red, (D) lateral and (E) basal views, the mandibles were removed to enhance the view of the of cranial base, arrows indicate absent bones or abnormal elements. (F) The mandible was shorter in *Spry1;Wnt1-Cre *embryos compared to WT controls. (G-H) Skeletal preparations of newborn mice, (G) dorsal and (H) basal views. Data presented are representative of five litters analyzed at this gestational age. Na: nasal capsule; nb: nasal bone; fn: front bone; bs: basisphenoid; eo: exooccipital; ip: interparietal bone; mx maxilla; md: mandible; ob: basioccipital; pa: parietal bone; ps: presphenoid; so: supraoccipital. (I) In utero MR images of WT and *Spry1;Wnt1-Cre *embryos at 14 dpc. The overview image shows 4 embryos in varying orientations and views. The enlarged view on the right side show (top) a WT embryo with normal facial and cardiac development and (bottom) a *Spry1;Wnt1-Cre *embryo with severe facial malformations, enlarged heart and defective cardiac outflow tract. Images were obtained with a RARE pulse sequence (TE 39.8 ms, TR 2571 ms, FOV 35 × 35 mm, matrix 256 × 256, slice thickness 1 mm (total of 7 slices), 3 averages, total scan time 4 min. 6 sec).

Severe facial clefting was detected at E16.5 in *Spry1;Wnt1-Cre *embryos but not their Cre-negative littermates (Figure 2A-C). Skeletal preparations of E16.5 *Spry1;Wnt1-Cre *embryos revealed hypoplastic and malformed bones and cartilage of the head (Figure 2D, E) and neck (data not shown). The maxilla was incomplete and malformed (Figure 2E) and the mandible was smaller (Figure 2F). Skeletal preparations of E18.5 *Spry1;Wnt1-Cre *embryos reveals a complete absence of the frontal and nasal bones, whereas the parietal, interparietal and occipital bones that are not derived from neural crest, formed normally (Figure 2G, H). In addition, the premaxilla and maxilla were malformed or absent and the zygomatic arch was poorly developed. Defects in development were also detected by MRI at E14 and included in addition to the externally visible craniofacial defects, but also defects in cardiac development including dilatation of cardiac chambers and an outflow tract defect (Figure 2I).

### Conditional expression of Spry1 inhibits palatogenesis

**Figure 3:**
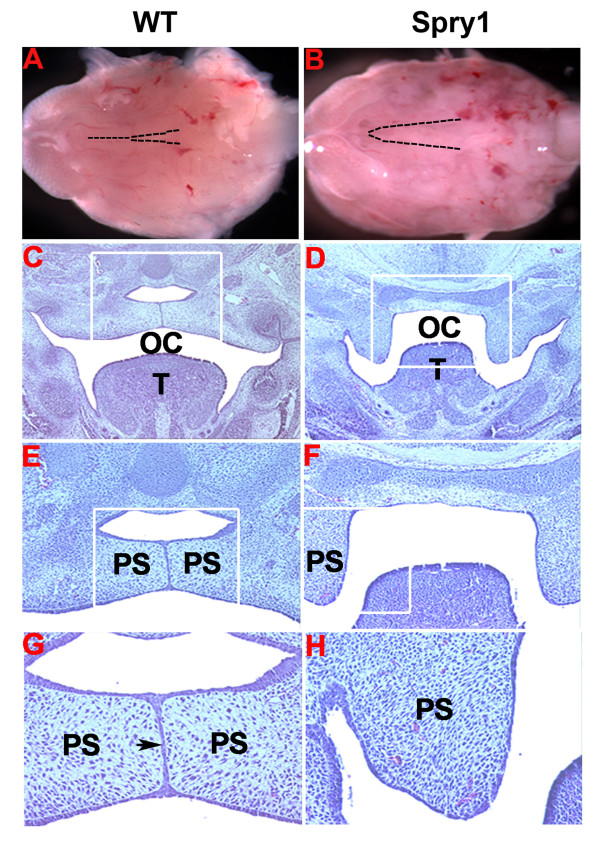
***Spry1;Wnt1-Cre *embryos exhibit cleft palate**. (A, B) The developing mandible and tongue were removed from E14.5 embryos to show the developing medial epithelial seam of the palatal shelves in WT but not in *Spry1;Wnt1-Cre *(Spry1) embryos (dash line indicated), (C-H) cross sections of E16.5 embryo heads stained with hematoxylin and eosin. (C, D) Low magnification shows the fused palate and the separated nasopharynx and oral cavity in WT control, but not in *Spry1;Wnt1-Cre *embryos (from original 10 ×), (E, F) high magnification from the boxed areas in C and D (from original 10 ×), (G, H) high magnification from the boxed areas of E and F (from original 20 ×). OC: oral cavity; PS: palatal shelves; T: tongue. Data are representative of four litters analyzed as this stage.

The failure of fusion of the secondary palate of *Spry1;Wnt1-Cre *embryos was evident by E14.5 when Cre-negative littermate controls showed normal fusion of the palatal shelves (Figure 3A, B). We compared cross-sections of E16.5 *Spry1;Wnt1-Cre *mutant embryonic heads with Cre-negative littermate control embryos. At E16.5 there was a failure of the elevation of the palatal shelved in *Spry1;Wnt1-Cre *embryos (Figure 3D, F, and 3H), while the Cre-negative control littermates showed elevation and fusion of the palatal shelves (Figure 3C, E, and 3G).

### Conditional expression Spry1 inhibits proliferation and increases apoptosis in neural crest and neural crest-derived structures

**Figure 4:**
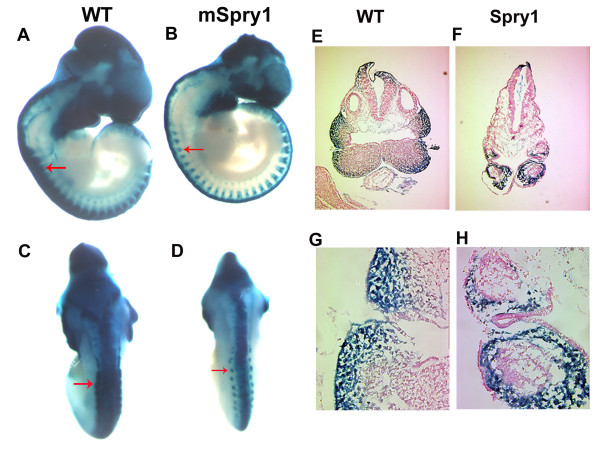
***Spry1;Wnt1-Cre *embryos have reduced β-gal staining in neural crest-derived structures**. (A-D) Whole mount β-gal staining, (A, B) lateral view to show neural crest cells (blue) have contributed to the branchial arches and lateral mesoderm both in *Spry1;Wnt1-Cre *(Spry1) and WT control mice, however the β-gal stained neural crest derivatives in Spry1 embryos were smaller than those of the controls (WT) (arrows indicated). (C, D) Dorsal view to show the similar intensity of β-gal staining at the ridge of the neural tube of *R26R;Wnt1-Cre *(WT) or *Spry1;R26R;Wnt1-Cre *embryos. (E-H) Cross sections of E9.5 whole mount β-gal stained embryos. (E, F) Low magnification to show intensity of β-gal staining in ridge of neural tube and brachial arches of WT and *Spry1;Wnt1-Cre *embryos. (G, H) High magnification to show the decreased cellular mass in the first branchial arch of *Spry1;Wnt1-Cre *embryos with reduced β-gal staining. Data are representative of three independent experiments.

**Figure 5:**
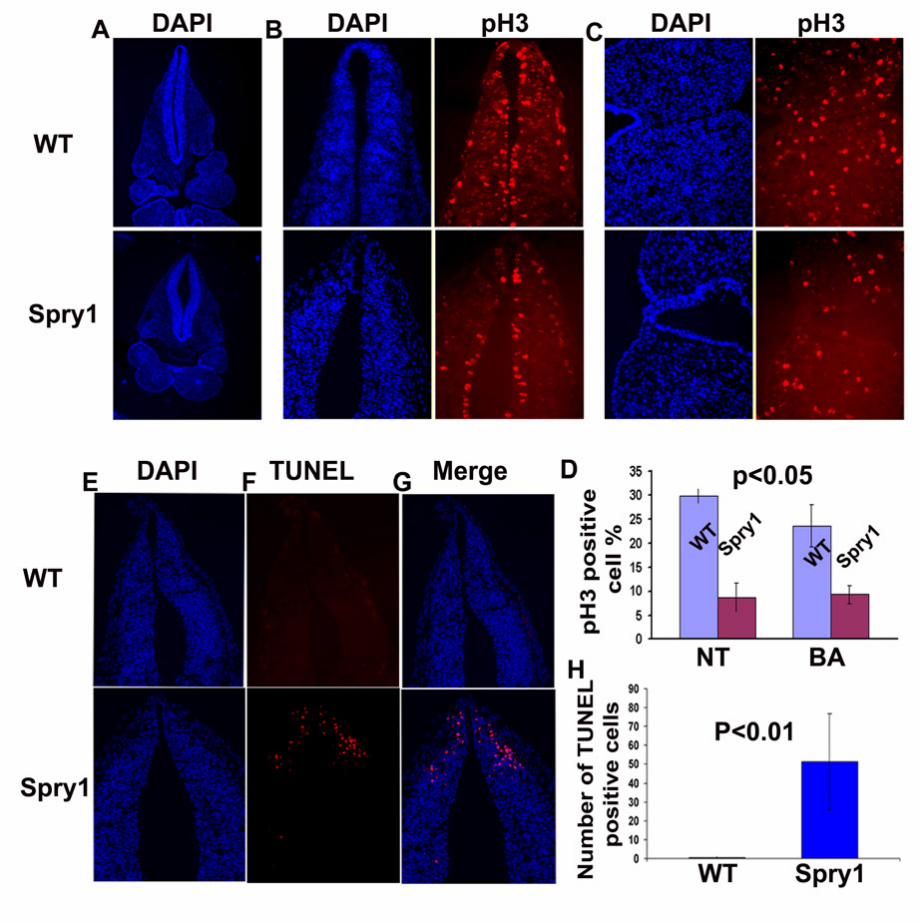
**Overexpression of Spry1 in neural crest-derived cells induced apoptosis and reduced cell proliferation**. (A-D) Phospho-histone H3 immunofluorescence staining of E10.5 embryo cross sections showed the reduced phospho-histone H3 positive cells in *Spry1;Wnt-Cre *(Spry1) embryos compared to Cre-negative littermate controls(WT) (from original 40×). Low magnification to show similarity of sections (from original 10×) (A), high magnification to show the decreased phospho-histone H3 positive cells in neural tube (B) and branchial arches (C) of *Spry1;Wnt1-Cre *mice compared to WT control (from original 40×). (D) Quantification of phospho-histone H3 positive cells. (E-G) TUNEL labeling shows an increase in apoptotic cells in the neural tube of *Spry1;Wnt1-Cre *embryos but not in control embryos (from original 40×). (H) Quantification of TUNEL labeled cells. NT: neural tube; BA: branchial arch. Data shown are representative of three independent experiments.

To gain insight into the possible mechanisms that contribute to the craniofacial defects observed in *Spry1;Wnt1-Cre *embryos we crossed the conditional *CAGGFP-Spry1 *mice with *R26R;Wnt1-Cre *transgenic mice to generate *Spry1;R26R;Wnt1-Cre *mutant embryos. Whole-mount β-gal staining (Figure 4A-D) and sections (Figure 4E-H) through E10.5 *Spry1;R26R;Wnt1-Cre *embryos revealed β-gal positive cells remained in the dorsal neural tube of both *Spry1;R26R:Wnt1-Cre *and *R26R;Wnt1-Cre *embryos at this stage even though most cranial neural crest cells have emigrated from this region. In addition, the branchial arches of *Spry1;R26R:Wnt1-Cre *embryos were smaller than that of the *R26R;Wnt1-Cre *control embryos, however β-gal-positive cells were present (Figure 4E-H). The distribution of β-gal-positive cells was also altered in *Spry1;R26R;Wnt1-Cre *embryos with reduced mesenchymal cells underlying the β-gal-positive cells. There was also reduced β-gal staining in the trunk of *Spry1;R26R;Wnt1-Cre *embryos (Figure 4C, D red arrows). Therefore, to investigate a possible mechanism responsible for facial clefting and mandibular hypoplasia, we investigated whether there were changes in cell proliferation or apoptosis that would account for the observed craniofacial defects. Cell proliferation as measure by phospho-histone H3 immunostaining, was reduced approximately 2-fold in the neural tube of E10.5 *Spry1;Wnt1-Cre *mutant embryos when compared to their littermate controls (Figure 5B, D). In addition, proliferation was reduced in the branchial arches of E10.5 *Spry1;Wnt1-Cre *embryos when compared to littermate controls in regions of both NCC-derived and underlying mesodermal cells (Figure 5C, D). We also investigated the possibility that apoptosis may have contributed to the defects observed in *Spry1;Wnt1-Cre *mutant embryos. For programmed cell death analysis, TUNEL staining was performed on sections of E10.5 embryos. Significant TUNEL staining was detected in sections through the anterior neural tube of *Spry1;Wnt1-Cre *mutant embryos, but not in similar sections from Cre-negative control littermates (Figure F,G,H). Together these data are consistent with the reduced pattern β-gal staining in *Spry1;R26R;Wnt1-Cre *embryos and suggest that induced Spry1 expression in Wnt1-expressing cell populations inhibits proliferation and increases apoptosis contributing to the anatomical defects.

### Forced expression of Spry1 in transgenic mouse embryos decreases the expression domains of craniofacial marker genes

**Figure 6:**
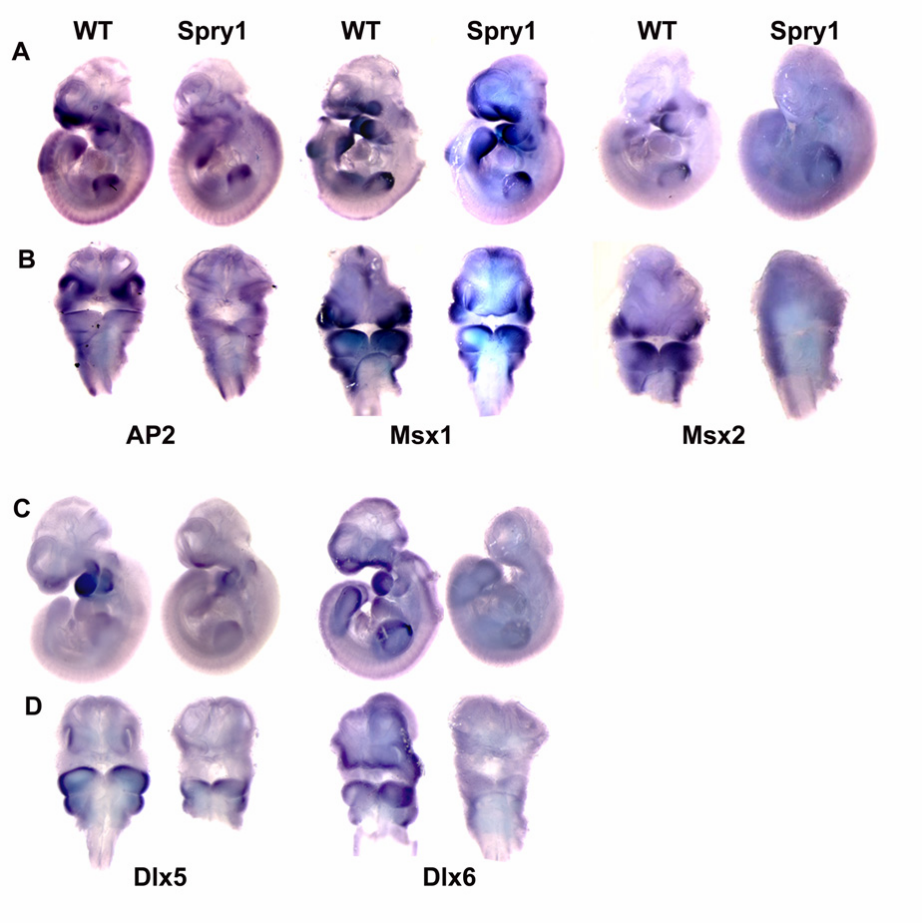
**Overexpression of Spry1 in neural crest cells decreased expression of craniofacial marker genes at E10.5**. (A, B) Overexpression of Spry1 decreases the expression domains of *AP2α*, *Msx1 *and *Msx2 *expression. (A) Lateral view to show the decreased expression of *AP2α*, *Msx1 *and *Msx2 *in the branchial arches of *Spry1;Wnt1-Cre *(Spry1) embryos, (B) frontal view to show the decreased expression of *AP2α*, *Msx1 *and *Msx2 *in maxilla and nasal pits of *Spry1;Wnt1-Cre *embryos. (C, D) Overexpression of Spry1 reduced expression domains of *Dlx5 *and *Dlx6*. (C) Lateral view to show the decreased expression of *Dlx5 *and *Dlx6 *in branchial arches of *Spry1;Wnt1-Cre *embryos. (D) Frontal view to show the decreased expression domains of *Dlx5 *and *Dlx6 *in maxilla and nasal pits of *Spry1;Wnt1-Cre *embryos compared to control (WT) mice. All in situ hybridization experiments were performed at least three times on *Spry1;Wnt1-Cre *embryos and their Cre-negative littermates. Color development time was equivalent for each experiment.

The proliferation, migration and differentiation of NCC are regulated by growth factor signaling pathways and downstream transcription factors. To investigate the effect of forced expression of Spry1 on the expression of genes crucial to craniofacial development and NCC differentiation we performed whole mount in situ hybridization on E10.5 *Spry1;Wnt1-Cre *embryos and their control littermates. *AP2α *is expressed in the neural crest cells of the dorsal neural tube during mammalian development [22]. We first examined *AP2α *expression in E10.5 *Spry1;Wnt1-Cre *embryos as we reasoned that expression of *AP2α *may be affected because *Spry1;Wnt1-Cre *embryos exhibited several abnormalities in common with *AP2α-/- *embryos. Whole mount in situ hybridization revealed that *AP2α *expression was greatly reduced in *Spry1;Wnt1-Cre *embryos compared to their control litter mates (Figure 6A and 6B). We next examined the expression of *Msx1 *and *Msx2 *in *Spry1;Wnt1-Cre *embryos. Mice that are homozygous null for both *Msx1 *and *Msx2 *exhibit severe craniofacial dysmorphology and a complete absence of the frontal bone [23]. *Spry1;Wnt1-Cre *embryos also exhibit a complete absence of the frontal bone; therefore we surmised that *Msx1 *and *Msx2 *expression would be reduced or absent. Our data indicate that forced expression of Spry1 in Wnt1-Cre expressing cells results in reduced domains of *Msx1 *and *Msx2 *expression in craniofacial structures, while expression in the limb buds remains intact albeit at a reduced level.

The homeobox genes *Dlx5 *and *Dlx6 *play important roles in craniofacial and limb development [24]. Mice that are null for both *Dlx5 *and *Dlx6 *exhibit severe craniofacial, axial, and appendicular skeletal abnormalities, resulting in perinatal lethality. Whole mount in situ hybridization of E10.5 *Spry1;Wnt1-Cre *embryos show that domains of expression of *Dlx5 *in the first and second branchial arches are greatly reduced. Similarly, *Dlx6 *expression domains were reduced in the first and second branchial arches. *Dlx5 *and *Dlx6 *expression in the limbs was variable but often reduced.

### Fgf8 expression is maintained in Spry1-expressing transgenic mice

**Figure 7:**
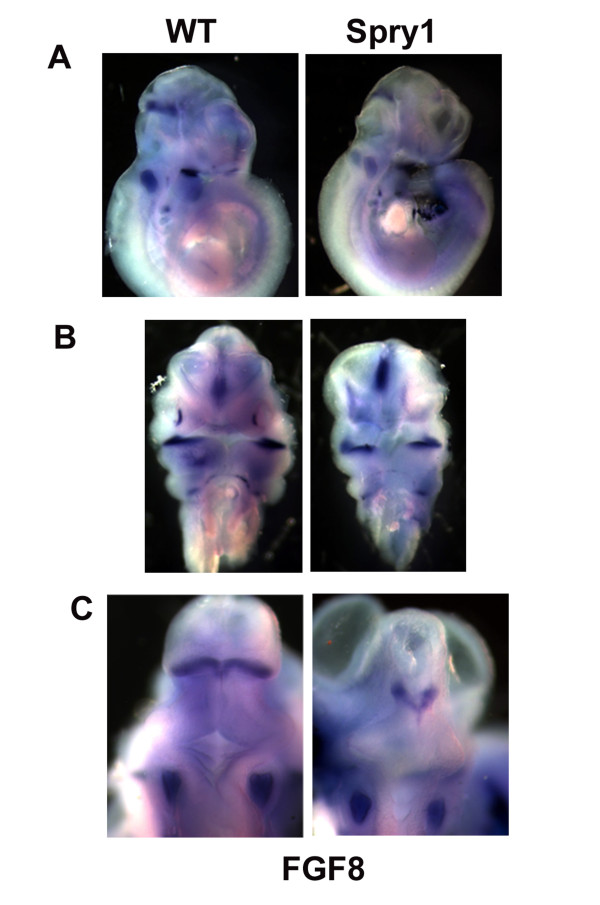
***fgf8 *expression is unaffected in *Spry1;Wnt1-Cre *embryos**. (A) Lateral view and (B) frontal view to show the similarity in pattern and intensity of *fgf8 *expression in both WT and *Spry1Wnt1-Cre *(Spry1) E10.5 embryos. (C) Dorsal view showing the similarity of expression of *fgf8 *in otic placode both in WT and *Spry1;Wnt1-Cre *embryos, however overexpression of Spry1 resulted in a reduced and abnormal midbrain-hindbrain boundary. Data are representative of three independent experiments.

FGFs and in particular fgf8 play an important role in neural crest development. Fgf8 expression in the ectoderm of the first and second branchial arch is induced by Shh signals from the foregut endoderm [7,25-27]. The branchial arch ectoderm-derived fgf8 in turn regulates the proliferation and differentiation of post-migratory NCC. To determine whether fgf8 expression was altered in *Spry1;Wnt1-Cre *embryos through a cell non-autonomous mechanism, we performed whole mount in situ hybridization with an fgf8 riboprobe (Figure 7). These data indicate that fgf8 expression remains intact in E10.5 *Spry1;Wnt-Cre *embryos when compared to their litter mate controls. Although there are some differences in the size and shape of the nasal placodes, the first branchial arch and isthmus of the midbrain-hindbrain boundary, the intensity of the fgf8 signal is similar between *Spry1;Wnt1-Cre *and control embryos. This data rules out the possibility that changes in fgf8 expression or availability account for the decrease in proliferation seen in the first arch of *Spry1;Wnt1-Cre *embryos (Figure 5C).

### Spry1;Wnt1-Cre embryos have cranial nerve patterning defects

**Figure 8:**
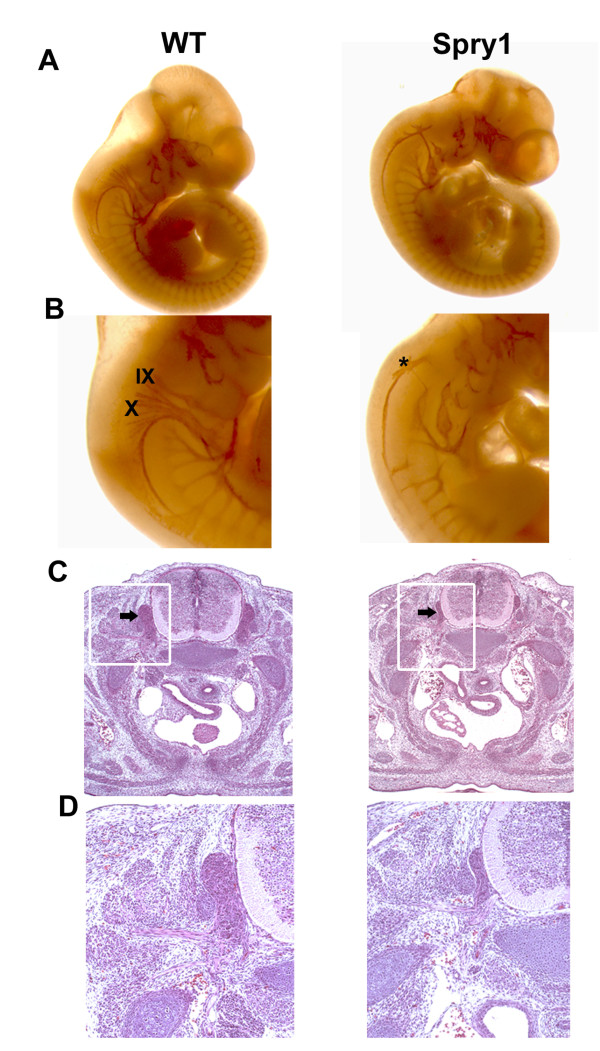
**Impaired cranial nerve development in *Spry1;Wnt1-Cre *embryos**. (A) Whole mount staining with neurofilament antibody at E10.5 shows smaller nerve filament bundles in *Spry1Wnt1-Cre *(Spry1) embyos compared to Cre-negative littermate controls (WT). (B) Higher magnification to show the defect in cranial nerves IX and X in *Spry1;Wnt1-Cre *transgenic embryos (* indicates misplaced cranial nerves). (C, D) Hematoxylin and eosin staining of E16.5 cross sections to show the smaller dorsal root ganglia in *Spry1;Wnt1-Cre *embryos compared to littermate controls (arrow indicated). (D) High magnification from boxed areas in C. IX: glossopharyngeal nerve; X: vagus nerve. Six *Spry1;Wnt1-Cre *and six control embryos representing two litters were analyzed.

NCC derived from rhombomeres 2, 4, 6, and 7 contribute to the formation of the cranial nerves [22,28]. To determine the effect of conditional expression of Spry1 in Wnt1-Cre expressing cells on cranial nerve morphogenesis, E10.5 *Spry1;Wnt1-Cre *embryos were immunostained with a neurofilament-M antibody. Results reveal abnormalities in several cranial nerves, with the most severe defects in cranial nerves IX (glossopharyngeal) and X (vagus) (Figure 8A and 8B). In *Spry1;Wnt1-Cre *embryos there is a disruption of branching of cranial nerves IX and X and a displacement from their normal position (Figure 8B). To determine whether these defects persist at later stages of development we performed H&E staining on sections of E16.5 *Spry1;Wnt1-Cre *embryos and their Cre-negative littermates. Transverse sections posterior to the otic placode reveals severe hypoplasia of dorsal root ganglia (Figure 8C and 8D).

### Cardiovascular malformations in Spry1;Wnt1-Cre transgenic mice

**Figure 9:**
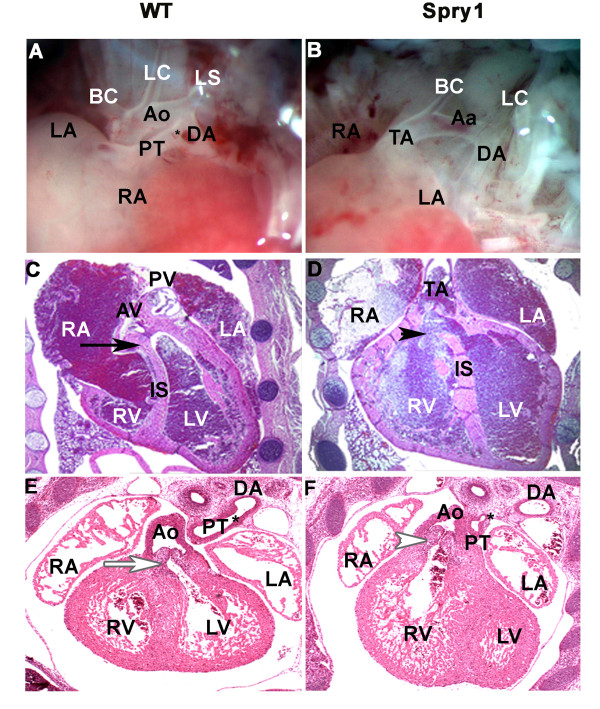
***Spry1;Wnt1-Cre *embryos exhibit cardiac outflow tract malformations and associated cardiac defects**. (A, B) Cre-negative littermate control (WT) and *Spry1;Wnt1-Cre *(Spry1) embryos at E18.5; gross photos of heart. A, shows WT with normal outflow tract and aortic arch architecture, and B shows abnormal outflow tract architecture with a persistent truncus arteriosus (TA) and aortic arch anomalies. (C, D) WT and *Spry1;Wnt1-Cre *E18.5 embryos, coronal sections through the thoracic cage stained with H&E. (C) normal outflow tract with aortic valve (AV) and pulmonary valve (PV) and an intact interventricular septum (black arrow). (D) *Spry1;Wnt1-Cre *enbryo shows a persistent truncus arteriosus (TA) which overrides the interventricular septum (IS) with a membranous ventricular septal defect (black arrowhead). (E, F) WT control and *Spry1;Wnt1-Cre *E14.5 embryos sectioned through the thoracic cage. (E) WT normal cardiac architecture. (White arrow denotes left ventricular outflow). (F) Shows a double outflow right ventricle with the aorta (Ao) arising from the right ventricle (white arrowhead) and the pulmonary outflow tract, which is narrowed with a malformed pulmonary valve and ventricular septal defect (not shown). LV-left ventricle, RV-right ventricle, RA-right atrium, LA-left atrium, Ao-aorta, PT-pulmonary trunk, DA-dorsal aorta, TA-truncus arteriosus, Aa-aortic arch, PV-pulmonary valve, AV-aortic valve, BC-brachiocephalic artery, LC-left common carotid artery, LS-left subclavian artery, Asterix *-ductus arteriosus. Data are representative of six embryos from each group (WT and Spry1) from two independent litters.

MRI imaging of E14 embryos (Figure 2) prompted us to examine the hearts of E18.5 and E14.5 *Spry1;Wnt1-Cre *embryos. These analyses revealed outflow tract malformations including a persistent truncus arteriosus and double outflow right ventricle (DORV) and their associated cardiac defects (Figure 9). Histological examination of these hearts revealed failure of the outflow tract to septate into two distinct outflows resulting in a persistent truncus arteriosus. The truncus, which overrode the interventricular septum, had only one valvular structure with three leaflets and there was an associated membranous ventricular septal defect (VSD). The pulmonary trunk arose just distal the truncal valve on the left lateral side. The right ventricular outflow tract connection to the truncus was shifted to the right and pointed towards the midline in comparison to the WT in which the flow was directed towards the left side. The left ventricular outflow tract connected into the truncus in a similar configuration with the WT with the direction of flow pointing towards with a right. In Figure 9F, a DORV was the result of a malrotated/malaligned heart with a dextroposed aorta arising off the right ventricle with an associated membranous VSD. In addition, the pulmonary outflow tract was narrowed with a stenotic hypoplastic pulmonary valve, which lacked distinct leaflets. The pulmonary trunk connected to a patent ductus arteriosus ensuring an adequate blood supply to the pulmonary circulation via the aorta. In addition, there were observed aortic arch anomalies in these hearts as seen in Figure 9B; no distinct left subclavian artery was visualized.

**Figure 10:**
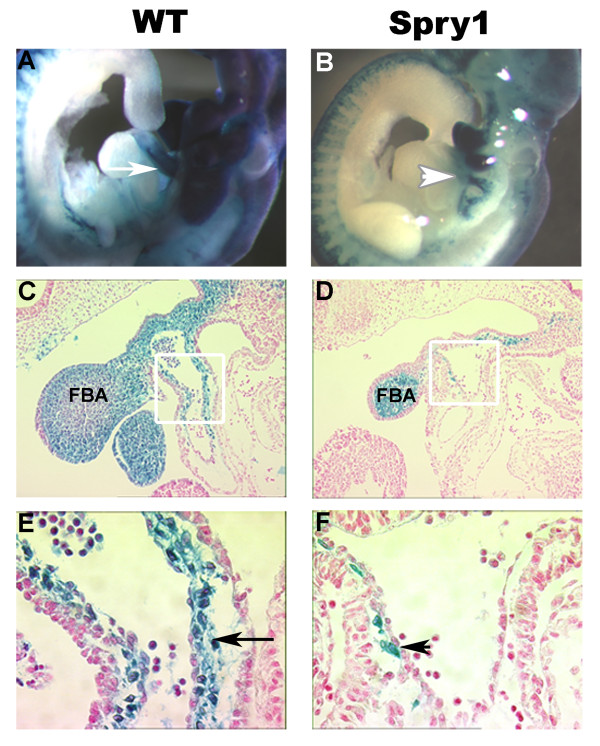
***Spry1;Wnt1-Cre *embryos show outflow tract defects at E9.5**. (A, B) Whole mount β-gal staining of Cre-negative littermate control and *Spry1;Wnt1-Cre *E9.5 embryos. (A) WT shows intense β-gal staining of the outflow tract (white arrow) and branchial arches, indicating cells of NC origin. (B) *Spry1;Wnt1-Cre *E9.5 embryo, shows variable β-gal staining of the pharyngeal arches and reduced staining of the outflow tract (white arrowhead). (C, D) Sagittal sections of whole mount embryos with nuclear fast red counter-staining. (C) WT shows normal distribution of cardiac NCC within the outflow tract, with β-galactosidase positive NCC cells extending down to the bulbis cordis. Panel D, *Spry1;Wnt1-Cre *reveals outflow tract with reduced β-galactosidase positive NCC cells. In addition the first branchial arch, mandibular component, is greatly reduced in size relative to the WT. The outflow tract in *Spry1;Wnt1-Cre *embryos is shortened and does not adopt the spiral configuration as seen in the WT. (E,F) High power images of C,D; white boxed areas indicate field of view. (E) WT, black arrow indicates cardiac NCC contributing cardiac mesenchyme. (F) *Spry1;Wnt1-Cre *embryo, black arrowhead notes paucity of NCC in cardiac mesenchyme. FBA: first branchial arch, mandibular component. Data are representative of six embryos from each group.

To analyze neural crest contributions to cardiac development in *Spry1;Wnt1-Cre *embryos, we examined histological sections taken from whole mount E9.5 *Spry1:R26R;Wnt1-Cre *embryos or *R26R;Wnt-Cre *littermates (Figure 10). Presumptive NCC marked by β-Gal staining in Figure 10A showed strong staining in the branchial arches and the outflow tract of control embryos. In contrast, *Spry1;R26R;Wnt1-Cre *embryos (Figure 10B) showed variable staining in the branchial arches and greatly diminished staining in the outflow tract. Histological sections (Figure 10C-F) revealed the first branchial arch was hypoplastic in the mutant vs. the control. The control embryos showed β-gal positive NCC colonizing the cardiac mesenchyme throughout the outflow tract and down into the bulbis cordis (Figure 10C, E). The mutant showed a paucity of β-gal positive neural crest cells colonizing the cardiac mesenchyme with a near total absence in the bulbis cordis. The outflow tract of the mutant was shortened and poorly rotated in comparison to the WT, which was elongated with a more spiral configuration. The lack of sufficient numbers of β-gal positive NCC colonizing the cardiac mesenchyme resulted in abnormal cardiac morphogenesis with the failure of the outflow tract to elongate normally, undergo normal cardiac looping, which as a consequence altered the rotation, alignment and septation of the outflow tract. Septation most likely did not occur due to the failure of the formation of the aorticopulmonary septum whose formation is critically dependent upon sufficient numbers of cardiac NCC colonizing and proliferating in the cardiac mesenchyme. DORV was a consequence of the malrotation and malalignment of the outflow tract, which was not positioned into its normal configuration between the atrioventricular valves. Cardiac NCC, in conjunction with the cells of the primary and secondary heart fields, are essential for normal formation of the endocardial cushions and conotruncal cushions. Deficiencies in these structures, which are dependant on cardiac NCC proliferation, signaling and interaction with the primary and secondary heart field cells, most likely led to the observed cardiac defects.

## Discussion

Our previous studies have revealed an important role for Spry1 in endochondral bone formation and chondrogenesis [21]. We undertook the present study to determine the role of Spry1 in craniofacial development. Previous studies using gene-targeting strategies revealed that targeted deletion of *Spry1 *[17] did not produce a craniofacial phenotype, and deletion of *Spry2 *produced defects in the inner ear [15] and dentition [16], however deletion of *Spry2 *and *Spry4 *results in several abnormalities including facial clefting and limb defects [18]. In furtherance of these studies, we used *Spry1 *transgenic mice to gain additional insight into the role of Spry1 in craniofacial development. To gain insight into the role of Spry1 in development of NCC-derived structures, we used a floxed transgenic allele of *Spry1 *[21], and induced its expression in NCC by Cre-mediated recombination driven by the *Wnt1 *promoter [20]. Mutant embryos died perinatally from multiple defects including severe facial clefting and cardiovascular defects including persistent truncus arteriosus and ventricular septation defects. We also observed hypoplasia of the thymus and thyroid glands (Kilgallen and Friesel, data not shown). Our results with *Spry1;Wnt1-Cre *embryos are consistent with insufficient neural crest-derived cells populations for normal craniofacial and cardiac morphogenesis. Our data indicate that increased apoptosis and decreased cell proliferation likely cause the NCC insufficiency. Although our analysis was performed at an embryonic stage where most cranial neural crest have emigrated from the neural tube, Wnt1-Cre mediated β-gal staining was still evident in *Spry1;R26R;Wnt1-Cre *embryos in the dorsal neural tube at this stage. This suggests that some residual neural crest cells or neural crest-derived cells remained in this region suggesting that decreased proliferation and increased apoptosis in the dorsal neural tube may be attributable to this population of β-gal positive cells.

In *Spry1;R26R;Wnt1-Cre *transgenic embryos β-gal positive cells were present in the branchial arches, and the cardiac region, however the number of β-gal positive cells, and the overall size of the branchial arches and neural crest-derived cardiac structures was greatly reduced. Immunostaining for phospho-histone H3, a marker of proliferation, and TUNEL assays revealed decreased proliferation and increased apoptosis respectively in the branchial arches of *Spy1;Wnt1-Cre *embryos but not their Cre-negative littermates suggesting that the increase in NCC apoptosis is a contributor to the observed craniofacial defects. Because fgf8 expression was essentially normal in *Spry1;Wnt1-Cre *embryos, fgf8 availability is likely not a factor in decreased NCC survival. Furthermore, we cannot rule out the possibility that the β-gal positive cells in the branchial arches and outflow tract did not undergo recombination of the Spry1 transgene, which may account for the β-gal positive cells that are present in this region. However, the increase in apoptosis and decrease in proliferation in this region suggest that Spry1 expression affected development of these structures possibly by affecting the reciprocal signaling between the NCC-derived mesoderm and the overlying ectoderm.

Palate development is a multistep process that involves the growth, elevation and midline fusion of the palatal shelves. The palatal shelves are comprised of NCC-derived ectomesenchyme and pharyngeal ectoderm [1,20,29]. The growth and development of the palate is controlled by several growth factors including members of the TGF-β family and members of the FGF family. Conditional loss-of-function of *Tgfbr2 *in NCC of *Tgfbr2*^*fl/fl*^; *Wnt1-Cre *mutant mice results in cleft palate [29]. Mice carrying a large deletion of chromosome 14 (Pub36-/-), a region that contains the *Spry2 *gene exhibit cleft palate, excessive cell proliferation and up regulation of FGF target genes including *Msx1*, *Etv5 *and *Barx1 *[19]. Interestingly, targeted disruption of *Spry2 *did not phenocopy the megabase deletion in chromosome 14; however a BAC *Spry2 *transgene expressing reduced levels of *Spry2 *completely rescued the facial clefting and cleft palate phenotype in *Pub36-/- *mice [19]. These data suggest that palate development is sensitive to *Spry2 *gene dosage. Our data are consistent with this notion. *Spry1 *and *Spry2 *have overlapping domains of expression during development and current data suggest that they may be functionally redundant in regulating FGF signaling [13]. Here we show that Spry1 over expression in neural crest derivatives partially phenocopies the palate defect of the *Pub36-/- *mutation. Together, these data suggest that normal palate development is in part dependent upon proper growth factor signaling thresholds, and that *Spry1 *and *Spry2 *play a key role in regulating these thresholds. Whether the roles of *Spry1 *and *Spry2 *are functionally redundant in palate development remains to be determined using tissue-specific loss-of-function approaches targeting two or more *Spry *family members in neural crest in vivo.

In addition to controlling palate development, TGFβ receptors and tyrosine kinase receptors (RTK) regulate the development of the calvarial bones of the skull that are derived from NCC [1,6,30-32]. *Spry1;Wnt1-Cre *mice show craniofacial and cardiac phenotypes that are very similar to *Pdgfrα*^*fl/fl*^; *Wnt1-Cre *embryos including facial clefting, and aortic arch defects [30]. The similarity in phenotypes between a loss-of-function *Pdgfrα *mutant and a gain-of-function Spry1 mutant are consistent with the notion that Spry 1 inhibits signaling downstream of RTKs. While the phenotypes of *Spry1;Wnt1-Cre *and *Pdgfrα*^*fl/fl*^; *Wnt1-Cre *embryos are similar, *Pdgfrα*^*fl/fl*^; *Wnt1-Cre *embryos did not show any changes in proliferation or apoptosis and the authors speculated that the defects were due to defects in NCC differentiation [30]. Conversely, *Spry1;Wnt1-Cre *embryos showed decreased proliferation and increased apoptosis in NNC derived structures. While it is likely that increased proliferation and decreased apoptosis in NCC of *Spry1;Wnt1-Cre *embryos contributes to the phenotype, it is also possible that similar to the *Pdgfrα*^*fl/fl*^; *Wnt1-Cre*, *Spry1;Wnt1-Cre *have defects in differentiation. *Spry1;Wnt1-Cre *mice also share phenotypic similarities to *Alk5*^*fl/fl*^; *Wnt1-Cre *mice including cardiac defects [31] and craniofacial defects including cleft palate [32]. The *Alk5*^*fl/fl*^; *Wnt1-Cre *craniofacial defects are more severe in that they lack nasal and frontal bones and, parietal bones, whereas *Spry1;Wnt1-Cre *embryos lacked frontal and nasal bones but had nearly normal parietal bones. Whether Spry1 directly influences PDGFRα and Alk5 signaling in NCC directly will require further study.

Forced expression of Spry1 in Wnt1-expressing cells was also associated with defects in the development of cranial nerves including the glossopharyngeal nerve (IX) and the vagus nerve (X). Hypoplastic and patterning abnormalities of cranial nerves was revealed by immunostaining with neurofilament antibodies. Migrating Sox-10-expressing NCC contribute to cranial nerves IX and X, and these cells are reduced and their migration and guidance are defective in *Hox3a*^-/-^[33], *Fbln1*^-/-^[34], and *Msx1*^-/-^*;Msx2*^-/- ^mice. It is likely the defects in cranial nerves in *Spry1;Wnt1-Cre *embryos are due to a combination of reduced NCC proliferation or survival or altered responses to local guidance cues due to forced expression of Spry1.

*Spry1;Wnt1-Cre *embryos die perinatally due to craniofacial and cardiac defects including persistent truncus arteriosus and aortic pulmonary trunk abnormalities. Fate mapping studies using *Spry1;R26R;Wnt1-Cre *mice show that NCC correctly migrate into the branchial arches. It is likely that NCC insufficiency due to decreased proliferation and increased apoptosis in this region is the cause for the failure of formation of the aorticopulmonary septum, resulting in an overriding truncus arteriosus and DORV.

## Conclusion

Our results show that Spry1 is expressed in neural crest and neural crest derived craniofacial structures. Forced expression of Spry1 in Wnt1-Cre expressing cells resulted craniofacial and cardiac defects. Our data and that of others suggest that appropriate Spry1 levels are important to correct patterning of neural crest derived structures including bones of the face and the cardiac outflow tract. The similarity of the *Spry1;Wnt1-Cre *embryonic phenotype to the phenotypes of *Pdgfrα*^*fl/fl*^; *Wnt1-Cre *are consistent with Spry1 inhibiting signaling downstream of RTKs. The similarity of the *Spry1;Wnt1-Cre *embryonic phenotype to that of *Alk5*^*fl/fl*^; *Wnt1-Cre *embryos suggests a possible interaction of Spry1 with the Alk5 pathway.

## Methods

### Generation of *Spry1;Wnt1-Cre *mutant mice

*Wnt1-Cre *transgenic mice and *R26R *reporter mice have been described previously [29]. The generation of *CAGGFP-Spry1 *transgenic mice has been described elsewhere [21]. Briefly, the mouse Spry1 open reading frame was tagged with a myc/his epitope and cloned into the CAG-loxP-GFP-loxP vector (gift of J. Yoon). Transgenic mice were generated by pronuclear injection of the linearized plasmid, and transgenic mice screened by PCR of genomic DNA with GFP specific primers. The resulting transgenic mice were designated *CAGGFP-Spry1*. This transgenic line was maintained on a FVB genetic background. For lineage tracer analysis, *CAGGFP-Spry1 *mice were crossed with *R26R *mice. Mice that were positive for both GFP and β-galactosidase were then crossed with *Wnt1-Cre *mice. These mice carry CNC cells labeled with β-galactosidase before CNC cells begin to migrate out of the neural tube [29]. Additional Spry1 expression studies were performed on *Spry1*^*lacZ*/+ ^mice were obtained from the Mutant Mouse Regional Resource, University of California, Davis, and recently described [35]. Detection of β-galactosidase activity (β-gal) activity on whole embryos and tissue sections was carried by using standard procedures [21]. To over express Spry1 in neural crest cells *CAGGFP-Spry1 *mice were crossed with *Wnt1-Cre *mice, and double transgenic mice were identified by PCR of genomic DNA from either tails or placenta using specific primer for GFP and Cre.

All mice were housed in a pathogen-free environment, under light, temperature, and humidity controlled conditions. The Maine Medical Center Research Institute Institutional Animal Care and Use Committee approved all procedures involving animals.

### Skeletal preparations

Skeletal preparations were performed as described [29]. Briefly, timed pregnant females or newborn mice were euthanized by asphyxiation in CO_2_. Embryos and neonates were skinned, eviscerated, and fixed in 95% ethanol. The skeletons were stained with alcian blue, cleared in 1% KOH, and counterstained with alizarin red.

### Magnetic resonance imaging

Magnetic resonance (MR) images were obtained with a BRUKER PharmaScan 7 T, 300 MHz scanner using a RARE 8 pulse sequence with the following parameters: TE 39.8 ms, TR 2571 ms, FOV 35 × 35 mm, Matrix 256 × 256, Slice 1 mm (total of 7 slices), 3 averages, total scan time 4 min 6 sec. Pregnant female mice were maintained under anesthesia using 2% isoflurane, a slightly higher percentage than used in other scans to minimize embryonic movement and to allow non-breathing gated image acquisition. The total anesthesia time was less than 30 min and the pregnant females recovered normally from the procedure. The orientation of the image slices was chosen such that two embryos could be imaged in the sagittal view.

### Histology and in situ hybridization

For histological analysis, embryos were fixed in 4% paraformaldehyde, and were either embedded in OCT, and serial 7 μm-frozen sections were prepared, or embryos were embedded in paraffin and sectioned using standard procedures. For general morphology, deparaffinized sections were stained with hematoxylin and eosin using standard procedures.

For whole-mount in situ hybridization, plasmids were linearized with appropriate restriction enzyme; digoxygenin-labeled riboprobes were generated using a kit (Roche) according to manufacturer's protocol. Fgf8 probe was from P.H. Crossley, *Msx1*, *Msx2*, *Dlx5*, and *Dlx6 *were from Yang Chai, *AP-2 *was from Trevor Williams. In situ hybridization was processed according to established protocols. Briefly, embryos were washed with PTW (PBS + 0.1% Tween-20), treated with 10 μg/ml proteinase K briefly, prehybridized at 65°C, hybridized with indicated antisense probe at 65°C for overnight, then detected with alkaline phosphatase-conjugated anti-digoxygenin antibody (Roche), and developed with BM purple (Roche).

### X-gal Staining

Mouse embryos at E9.5 or E10.5 were fixed in 4% paraformaldehyde for 10 mins, washed in PBS, and stained in X-gal solution at 37°C overnight. After staining, embryos were refixed and embedded in either OCT or paraffin; 7 μm cryostat sections were taken for microscopic analysis.

### Proliferation and apoptosis assays

Embryos were collected at E9.5 or E10.5, fixed in 4% paraformaldehyde and embedded in OCT, and serial 7 μm sections were prepared for proliferation or apoptosis analysis. Immunofluorescent staining using anti-phosphor-Histone3 (Ser10) antibody (Upstate) was performed, phosphor-H3 positive cell was quantified and the proliferation rates were expressed as a percentage of total cells. For TUNEL labeling, the fluorescent in situ Cell Death Detection kit (Roche) was used according to the manufacturer's instructions, and the number of apoptotic cells per section was quantified.

## Authors' contributions

XY carried out phenotypic and molecular analyses of Spry1;Wnt1Cre embryos and manuscript editing, SK performed histological analysis of cardiovascular, palatal, and neural defects, VA performed developmental embryonic expression analysis of Spry gene expression, DBS edited the manuscript and provided input on experimental design, IP performed MRI analyses and analyzed the data, RF supervised the project, designed experiments, obtained grant support and finalized the manuscript. All authors read and approved the manuscript.

## References

[B1] Chai Y Maxson RE Jr Recent advances in craniofacial morphogenesis Dev Dyn 2006 235 2353 75 10.1002/dvdy.20833 16680722

[B2] Sauka-Spengler T Bronner-Fraser M A gene regulatory network orchestrates neural crest formation Nat Rev Mol Cell Biol 2008 9 557 68 10.1038/nrm2428 18523435

[B3] Neilson KM Friesel RE Constitutive activation of fibroblast growth factor receptor-2 by a point mutation associated with Crouzon syndrome J Biol Chem 1995 270 26037 40 10.1074/jbc.270.16.9597 7592798

[B4] Britto JA Evans RD Hayward RD Jones BM From genotype to phenotype: the differential expression of FGF, FGFR, and TGFbeta genes characterizes human cranioskeletal development and reflects clinical presentation in FGFR syndromes Plast Reconstr Surg 2001 108 2026 39 discussion 2040-6. 10.1097/00006534-200112000-00030 11743396

[B5] Sarkar S Petiot A Copp A Ferretti P Thorogood P FGF2 promotes skeletogenic differentiation of cranial neural crest cells Development 2001 128 2143 52 11493535 10.1242/dev.128.11.2143

[B6] Sasaki T Ito Y Bringas P Jr Chou S Urata MM Slavkin H Chai Y TGF{beta}-mediated FGF signaling is crucial for regulating cranial neural crest cell proliferation during frontal bone development Development 2006 133 371 81 10.1242/dev.02200 16368934

[B7] Abu-Issa R Smyth G Smoak I Yamamura K Meyers EN Fgf8 is required for pharyngeal arch and cardiovascular development in the mouse Development 2002 129 4613 25 12223417 10.1242/dev.129.19.4613

[B8] Trokovic N Trokovic R Mai P Partanen J Fgfr1 regulates patterning of the pharyngeal region Genes Dev 2003 17 141 53 10.1101/gad.250703 12514106 PMC195961

[B9] Hacohen N Kramer S Sutherland D Hiromi Y Krasnow MA sprouty encodes a novel antagonist of FGF signaling that patterns apical branching of the Drosophila airways Cell 1998 92 253 63 10.1016/S0092-8674(00)80919-8 9458049

[B10] Kramer S Okabe M Hacohen N Krasnow MA Hiromi Y Sprouty: a common antagonist of FGF and EGF signaling pathways in Drosophila Development 1999 126 2515 25 10226010 10.1242/dev.126.11.2515

[B11] Casci T Vinos J Freeman M Sprouty, an intracellular inhibitor of Ras signaling Cell 1999 96 655 65 10.1016/S0092-8674(00)80576-0 10089881

[B12] Thisse B Thisse C Functions and regulations of fibroblast growth factor signaling during embryonic development Dev Biol 2005 287 390 402 10.1016/j.ydbio.2005.09.011 16216232

[B13] Mason JM Morrison DJ Basson MA Licht JD Sprouty proteins: multifaceted negative-feedback regulators of receptor tyrosine kinase signaling Trends Cell Biol 2006 16 45 54 10.1016/j.tcb.2005.11.004 16337795

[B14] Minowada G Jarvis LA Chi CL Neubuser A Sun X Hacohen N Krasnow MA Martin GR Vertebrate Sprouty genes are induced by FGF signaling and can cause chondrodysplasia when overexpressed Development 1999 126 4465 75 10498682 10.1242/dev.126.20.4465

[B15] Shim K Minowada G Coling DE Martin GR Sprouty2, a mouse deafness gene, regulates cell fate decisions in the auditory sensory epithelium by antagonizing FGF signaling Dev Cell 2005 8 553 64 10.1016/j.devcel.2005.02.009 15809037

[B16] Klein OD Minowada G Peterkova R Kangas A Yu BD Lesot H Peterka M Jernvall J Martin GR Sprouty genes control diastema tooth development via bidirectional antagonism of epithelial-mesenchymal FGF signaling Dev Cell 2006 11 181 90 10.1016/j.devcel.2006.05.014 16890158 PMC2847684

[B17] Basson MA Akbulut S Watson-Johnson J Simon R Carroll TJ Shakya R Gross I Martin GR Lufkin T McMahon AP Sprouty1 Is a Critical Regulator of GDNF/RET-Mediated Kidney Induction Dev Cell 2005 8 229 239 10.1016/j.devcel.2004.12.004 15691764

[B18] Taniguchi K Ayada T Ichiyama K Kohno R Yonemitsu Y Minami Y Kikuchi A Maehara Y Yoshimura A Sprouty2 and Sprouty4 are essential for embryonic morphogenesis and regulation of FGF signaling Biochem Biophys Res Commun 2007 352 896 902 10.1016/j.bbrc.2006.11.107 17156747

[B19] Welsh IC Hagge-Greenberg A O'Brien TP A dosage-dependent role for Spry2 in growth and patterning during palate development Mech Dev 2007 124 746 61 10.1016/j.mod.2007.06.007 17693063 PMC2043129

[B20] Chai Y Jiang X Ito Y Bringas P Jr Han J Rowitch DH Soriano P McMahon AP Sucov HM Fate of the mammalian cranial neural crest during tooth and mandibular morphogenesis Development 2000 127 1671 9 10725243 10.1242/dev.127.8.1671

[B21] Yang X Harkins LK Zubanova O Harrington A Kovalenko D Nadeau RJ Chen PY Toher JL Lindner V Liaw L Overexpression of Spry1 in chondrocytes causes attenuated FGFR ubiquitination and sustained ERK activation resulting in chondrodysplasia Dev Biol 2008 321 64 76 10.1016/j.ydbio.2008.05.555 18582454 PMC2548288

[B22] Zhang J Hagopian-Donaldson S Serbedzija G Elsemore J Plehn-Dujowich D McMahon AP Flavell RA Williams T Neural tube, skeletal and body wall defects in mice lacking transcription factor AP-2 Nature 1996 381 238 41 10.1038/381238a0 8622766

[B23] Han J Ishii M Bringas P Jr Maas RL Maxson RE Jr Chai Y Concerted action of Msx1 and Msx2 in regulating cranial neural crest cell differentiation during frontal bone development Mech Dev 2007 124 729 45 10.1016/j.mod.2007.06.006 17693062 PMC2220014

[B24] Robledo RF Rajan L Li X Lufkin T The Dlx5 and Dlx6 homeobox genes are essential for craniofacial, axial, and appendicular skeletal development Genes Dev 2002 16 1089 101 10.1101/gad.988402 12000792 PMC186247

[B25] Creuzet S Couly G Le Douarin NM Patterning the neural crest derivatives during development of the vertebrate head: insights from avian studies J Anat 2005 207 447 59 16313387 10.1111/j.1469-7580.2005.00485.x PMC1571568

[B26] Crump JG Maves L Lawson ND Weinstein BM Kimmel CB An essential role for Fgfs in endodermal pouch formation influences later craniofacial skeletal patterning Development 2004 131 5703 16 10.1242/dev.01444 15509770

[B27] Withington S Beddington R Cooke J Foregut endoderm is required at head process stages for anteriormost neural patterning in chick Development 2001 128 309 20 11152630 10.1242/dev.128.3.309

[B28] Ishii M Han J Yen HY Sucov HM Chai Y Maxson RE Jr Combined deficiencies of Msx1 and Msx2 cause impaired patterning and survival of the cranial neural crest Development 2005 132 4937 50 10.1242/dev.02072 16221730

[B29] Ito Y Yeo JY Chytil A Han J Bringas P Jr Nakajima A Shuler CF Moses HL Chai Y Conditional inactivation of Tgfbr2 in cranial neural crest causes cleft palate and calvaria defects Development 2003 130 5269 80 10.1242/dev.00708 12975342

[B30] Tallquist MD Soriano P Cell autonomous requirement for PDGFRalpha in populations of cranial and cardiac neural crest cells Development 2003 130 507 18 10.1242/dev.00241 12490557

[B31] Wang J Nagy A Larsson J Dudas M Sucov HM Kaartinen V Defective ALK5 signaling in the neural crest leads to increased postmigratory neural crest cell apoptosis and severe outflow tract defects BMC Dev Biol 2006 6 51 10.1186/1471-213X-6-51 17078885 PMC1635039

[B32] Dudas M Kim J Li WY Nagy A Larsson J Karlsson S Chai Y Kaartinen V Epithelial and ectomesenchymal role of the type I TGF-beta receptor ALK5 during facial morphogenesis and palatal fusion Dev Biol 2006 296 298 314 10.1016/j.ydbio.2006.05.030 16806156 PMC1557652

[B33] Chisaka O Capecchi MR Regionally restricted developmental defects resulting from targeted disruption of the mouse homeobox gene hox-1.5 Nature 1991 350 473 9 10.1038/350473a0 1673020

[B34] Cooley MA Kern CB Fresco VM Wessels A Thompson RP McQuinn TC Twal WO Mjaatvedt CH Drake CJ Argraves WS Fibulin-1 is required for morphogenesis of neural crest-derived structures Dev Biol 2008 319 336 45 10.1016/j.ydbio.2008.04.029 18538758 PMC2965525

[B35] Thum T Gross C Fiedler J Fischer T Kissler S Bussen M Galuppo P Just S Rottbauer W Frantz S MicroRNA-21 contributes to myocardial disease by stimulating MAP kinase signalling in fibroblasts Nature 2008 456 980 4 10.1038/nature07511 19043405

